# Preliminary forensic assessment of the visualised fingerprints on nonporous substrates immersed in water using the green and optimised novel nanobio-based reagent

**DOI:** 10.1038/s41598-022-18929-8

**Published:** 2022-08-30

**Authors:** Aida Rasyidah Azman, Naji Arafat Mahat, Roswanira Abdul Wahab, Wan Azlina Ahmad, Dzulkiflee Ismail

**Affiliations:** 1 Department of Chemistry, Faculty of Science, Universiti Teknologi Malaysia, 81310 Skudai, Johor Malaysia; 2 Enzyme Technology and Green Synthesis Research Group, Faculty of Science, Universiti Teknologi Malaysia, 81310 Skudai, Johor Malaysia; 3 Centre for Sustainable Nanomaterials, Ibnu Sina Institute for Scientific and Industrial Research, Universiti Teknologi Malaysia, 81310 Skudai, Johor Malaysia; 4 Forensic Science Programme, School of Health Sciences, Universiti Sains Malaysia, 16150 Kubang Kerian, Kelantan Malaysia

**Keywords:** Enzymes, Nanobiotechnology

## Abstract

The discovery of forensic evidence (e.g. weapons) during forensic underwater investigations has seen an increasing trend. To date, small particle reagent (SPR) has been one of the routinely used methods for visualising fingerprints on wet, non-porous substrates. However, the long term use of SPR is detrimental to humans and environment due to the use of toxic chemicals. Although previously we have successfully developed and optimised a greener nanobio-based reagent (NBR), its suitable practical use in a more realistic scene (e.g. outdoor pond) was not evaluated. Therefore, this present research is aimed at (1) investigating the performance of NBR against the benchmark SPR in visualising fingerprints immersed in a natural outdoor pond and (2) evaluating the greenness of NBR against the analytical Eco-Scale. Results showed that the performance of the optimised NBR was mostly comparable (University of Canberra (UC) comparative scale: 0) with SPR at visualising fingerprints on three different non-porous substrates immersed in a natural outdoor pond. Observably, the NBR had higher preference towards aged fingerprints (up to 4 weeks of immersion). In addition, its greenness assessment revealed 76 points, indicating ‘excellent green analysis’. The findings gathered here further supported the practical use of the NBR in forensic investigations.

## Introduction

Over the years, fingerprints have been globally considered the benchmark for identifying a suspect, victim or witness for forensic investigations^1^. A unique identifier on its own, fingerprints consists of a series of random ridges and furrows formed within 6–25 weeks of human fetal development^2^, supporting the fact that no two individuals have the same fingerprints^1^. Forensically, fingerprints found at crime scenes can be of patent, plastic, or the most common, latent in nature^3^. Because the latter demands the use of suitable optical, physical, and/or chemical visualisation methods to be observable by naked human eyes, continuous method developments for visualising such prints have been frequently observed^4–6^. Nevertheless, the nature of the substrate (e.g. porosity and wetness) plays an important role in selecting the suitable method to visualise the latent prints.

It is important to highlight here that fingerprints are made up of eccrine (e.g. water, proteins and amino acids) and sebaceous (e.g. fatty acids and wax esters) constituents as well as other contaminants (e.g., cosmetics and bacteria)^7^. Expectedly, fingerprints left on wet substrates would only have a minute amount of non-water-soluble constituents, considering the water might have dissolved the water-soluble ones. Therefore, fingerprints subjected to destructive environments, like those found during underwater forensic investigations, further complicate the visualisation process. On the other hand, it has been reported that ‘criminals often seek a watery repository for weapons and other evidence of wrongdoing’, resulting in the alarming trend of accidents, drownings, violent crimes and homicides due to the increased use of recreational waterways^8^. For example, BBC News^9^ reported that about 80 weapons (including pistols and machine guns) were recovered from the Somerset river alone. The act of disposing of such forensic evidence is probably because the criminals ‘intended to confuse, hamper, or defeat investigative or forensic efforts to conceal their identity, their connection to the crime, or the crime itself’^10^. Contrary to popular assumption, recovering the fingerprints on such immersed objects are possible although the successful recovery rate is influenced by various factors (e.g. duration of exposure, physicochemical parameters of water and etc.)^8^.

Unlike the extensive published reports on novel and improved visualisation methods for dry substrates^11–14^, little can be said for methods on wet substrates. In this context, physical developer^15^, vacuum-^16^ and multi-metal depositions^17^, powder suspensions^18^ as well as small particle reagent (SPR)^19,20^ are the examples of methods for visualising fingerprints on wet substrates. Pertinently, physical developer and powder suspensions critically require the use of Synperonic® N and Triton™ X-100 (C-IOPS-09) as the surfactants, respectively. However, it has been established that the use of Synperonic® N has been banned by the European Union (EU) for industrial and marketing use under the EU directive 2003/53/EC (as cited in Thomas-Wilson, et al.^21^) due to its potential ecotoxicity. On the other hand, Downham, et al.^18^ highlighted the fact that Triton™ X-100 is classified under the Candidate List of Substances of Very High Concern, according to the European Comission (article 57 of the REACH Regulation). Hence, this necessitates the development of environmental benign alternatives for visualising latent fingerprints on wet substrates.

Being commercially available globally, SPR is commonly used for on-site visualisations by presumably capitalising on the lipids of fingerprints^1,22^. Despite the common usage of SPR, its mode of interactions underlying successful visualisation remains in dispute^23^. Typically, the commercially-available SPR (manufactured by Sirchie) is mainly made up of fine particles of either titanium dioxide (TiO_2_) (white), molybdenum (IV) sulphide (MoS_2_) (black) or invisible green (Sirchie proprietary fluorescent product) suspended in surfactant, and some, with the combination of ether and water^24–26^. However, such chemicals are often associated with numerous health risks and hazard threats to humans^27,28^ and the environment^29,30^. In actual fact, the International Agency for Research on Cancer^31^ has declared TiO_2_ as possibly carcinogenic to humans based on the empirical data from animal studies. The studies supported the notion that it may be due to the excessive production of intracellular reactive oxygen species^32^. It has to be highlighted here that although the on-site forensic investigators may not have been directly exposed to the fine particles considering that such suspended particles are relatively safer than in their powdery forms, the laboratory operators, on the other hand, would be repeatedly exposed to the harmful chemicals (possibly via inhalation) during the preparation process. Citing the New Jersey Department of Health^33^, “There may be no safe level of exposure to a carcinogen, so all contact should be reduced to the lowest possible level”, therefore the possibilities of suffering from such toxic exposures, although they have yet to raise alarming concerns, cannot be completely ruled out. On top of that, because the use of SPR requires rinsing with water (a few times)^34^, its long-term usage may prove detrimental to the ecosystem due to its bioaccumulation in the drainage. Despite its known perils, SPR continues to be the routinely used fingerprint visualisation methods, both in the laboratory and on-site crime scene investigations. Considering the safety as well as the well-being of the forensic investigators and the environment, developing a relatively safer and greener fingerprint visualisation reagent warrants forensic and environmental considerations.

Even though lipids are generally insoluble in water and being the preferred substrates for lipases, the practical use of lipases for tracing the minute amount of lipid constituents in fingerprints is still nascent. In this context, we had successfully developed^35^ and optimised^34^ a novel green nanobio-based reagent (NBR) by capitalising on the application of *Candida rugosa* lipase (CRL). It is important to mention that any newly-developed reagent for fingerprint visualisation must comply with the ‘Guidelines for The Assessment of Fingermark Detection Techniques’ prescribed by the International Fingerprint Research Group (IFRG)^36^ for ensuring its acceptance for forensic applications. Pertinently, the previously reported studies on the development and optimisation of NBR adhered to Phase 1 (proof-of-concept) and to a certain extent, Phase 2 (optimisation and comparison) of the guidelines. Not only a newly-developed fingerprint visualisation reagent needs to be specific and selective^34^, it must also be practical, simple (one solution) and rapid (within two minutes) and comparable with the performance of the commercially available SPR.

Thus, for assessing the robustness of the method in a more realistic situation, this present research is aimed at (1) investigating the performance of NBR against the benchmark SPR in visualising fingerprints immersed in a natural outdoor pond and (2) evaluating the greenness of the NBR against the prevailing analytical Eco-Scale.

## Methods

### Chemicals

While the CRL (type VII, ≥ 700 unit/mg solid) was purchased from Sigma-Aldrich (St. Louis, USA), acid-functionalised multiwalled carbon nanotubes (F-MWCNTs) and the commercially-available SPR mixture (SPR100 Small Particle Reagent-Dark, SPR100) were obtained from the Usains Holding Sdn. Bhd. (Penang, Malaysia) and Sirchie (Youngsville, USA), respectively. Additionally, both the acetone (99% purity) and potassium phosphate buffer (pH 7) were purchased from QRëC (Selangor, Malaysia).

### Experimental design

All the methods mentioned here were carried out in accordance with the relevant guidelines and regulations. Additionally, the ethical approval for executing this research was granted by the Research Ethics Committee (Human) of Universiti Sains Malaysia (USM/JEPeM/19010069). Despite the IFRG^36^ prefers the use of natural fingerprints, the fact that criminals may sweat and unconsciously touch their faces due to nervousness and agitation during the crime, and thereby producing sebaceous-rich fingerprints^35^, utilisation of both natural and groomed fingerprints investigated here proves relevant. In triplicates, five consenting, healthy, Malaysian donors (two males and three females aged between 25 and 37 years old) were asked to deposit both the natural and groomed split fingerprints on three different, non-porous substrates viz*.* glass slides, laminated plastics, and aluminium sheets.

**Figure 1:**
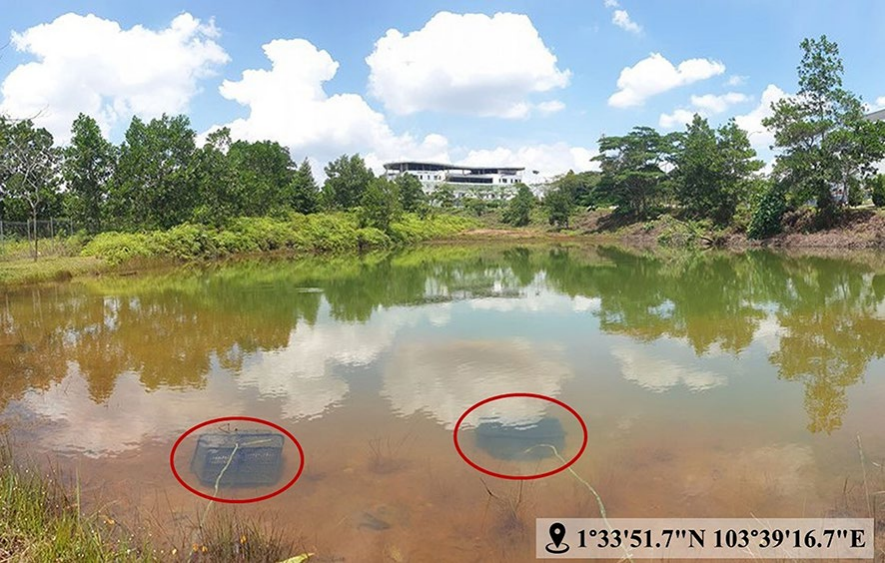
The actual field site of this research located within the Universiti Teknologi Malaysia Johor Bahru campus. The red circles marked the placement of the plastic baskets containing the non-porous substrates bearing fingerprints.

For obtaining natural fingerprints, the donors were instructed to strictly avoid (1) touching their T-zones (forehead, nose, and chin areas) and (2) washing their hands with/without soap for at least 30 min. On the other hand, groomed fingerprints were obtained via gentle rubbing of the thumbs across the T-zones; subsequently loading the thumbs with the non-water-soluble constituents. Using their right thumbs for both natural and groomed fingerprints, the donors were asked to place their thumbs at the joint of two identical non-porous substrates for 3 s, and consequently splitting the fingerprints into two equal halves. A gap of minimum 5 mins was maintained in between fingerprint depositions and the fingerprints were immersed in water upon completion of the fingerprint deposition that took place in about an hour or so prior to immersing them. Using nylon cable ties, the non-porous substrates bearing natural and groomed split fingerprints were separately secured onto a cylinder-like plastic mesh. For the purpose of safeguarding the fingerprint samples from aquatic animals (e.g. fishes and snails), the plastic mesh was laid in a slotted plastic basket (with side holes measuring at least 1 × 1 cm each) that was secured to the bank using raffia strings tied to metal tent pegs which were hammered into the ground. Subsequently, the basket was placed at the bed of the natural outdoor pond located within the Universiti Teknologi Malaysia Johor Bahru campus (1°33′51.7′′ N 103°39′16.7′′ E) for two and four consecutive weeks (Fig. 1). The in-situ physicochemical parameters of the natural outdoor pond (viz. pH, temperature, dissolved oxygen (DO) and turbidity) as well as total rainfall were recorded daily (around 12–2 PM) using handheld meters and a rain gauge, respectively. The visualised fingerprints were captured using a digital camera (Samsung SM-N960F). The performance of both reagents at visualising the wet fingerprints on non-porous substrates was assessed using the University of Canberra (UC) comparative scale^37^. It has to be mentioned here that the results were graded by two persons for ensuring the accuracy of the findings and avoiding bias. Additionally, the greenness of the NBR was also gauged using the prevailing analytical Eco-Scale suggested by Gałuska et al.^38^.

### Visualisation of the wet latent fingerprints using SPR and NBR as well as its quality assessment

Upon completion of the immersion interval, the left and right equal halves of natural and groomed split fingerprints were visualised using the SPR and NBR, respectively. While the SPR was used directly, the preparation of the NBR solution was done following the optimised and validated method using Response Surface Methodology suggested by Azman et al.^34^. The optimum conditions were; the amount of CRL (100 mg), amount of F-MWCNTs (75 mg) and immobilisation interval (5 h).

A small amount of the NBR was drawn using a plastic disposable dropper and dispensed directly onto the respective fingerprint deposition areas of the non-porous substrates and left incubated for two mins at room temperature (28–30°℃). As for SPR, the substrates bearing fingerprints were treated vertically, allowing the excess SPR to be drained away. Then, the non-porous substrates treated with both reagents were gently rinsed under running tap water and left to air dry. The visualised fingerprints were then digitally and permanently recorded using a camera and fingerprint tape lifter prior to appropriately grading them using the UC comparative scale (Table 1). While method A of the UC comparative scale refers to NBR, method B represents the SPR.

**Table 1:** The University of Canberra comparative scale used for assessing the performance of two reagent methods A and B at visualising wet latent fingerprints on non-porous substrates.

Score	Definition
+ 2	Half-impression developed by method A exhibits far greater ridge detail and/or contrast than the corresponding half-impression developed by method B
+ 1	Half-impression developed by method A exhibits slightly ridge detail and/or contrast than the corresponding half-impression developed by method B
0	No significant difference between the corresponding half-impressions
− 1	Half-impression developed by method B exhibits slightly ridge detail and/or contrast than the corresponding half-impression developed by method A
− 2	Half-impression developed by method B exhibits far greater ridge detail and/or contrast than the corresponding half-impression developed by method A

Method A represents NBR, method B is SPR.

### Greenness assessment of the novel NBR

For evaluating the greenness of the newly-developed NBR, the analytical Eco-Scale suggested by Gałuszka et al.^38^ was used. The assignments of penalty points were based on (1) amount and hazard of the chemicals, (2) required energy, (3) occupational hazard and (4) generation of waste. The cumulative penalty points were then deducted from the ideal green analysis value of 100. While score of more than 75 points falls under excellent green analysis, 50–75 points are considered acceptable; beyond which (less than 50), the analysis is deemed ‘inadequate green analysis’^38^.

### Ethics approval

Granted by the Research Ethics Committee (Human) of Universiti Sains Malaysia (USM/JEPeM/19010069).

### Consent to participate

The informed consent was obtained from all fingerprint donors included in the study.

## Results and discussions

### Forensic assessment of the novel NBR for visualising latent fingerprints immersed in a natural outdoor pond

#### Physicochemical parameters of the natural outdoor pond and total daily rainfall

**Figure 2:**
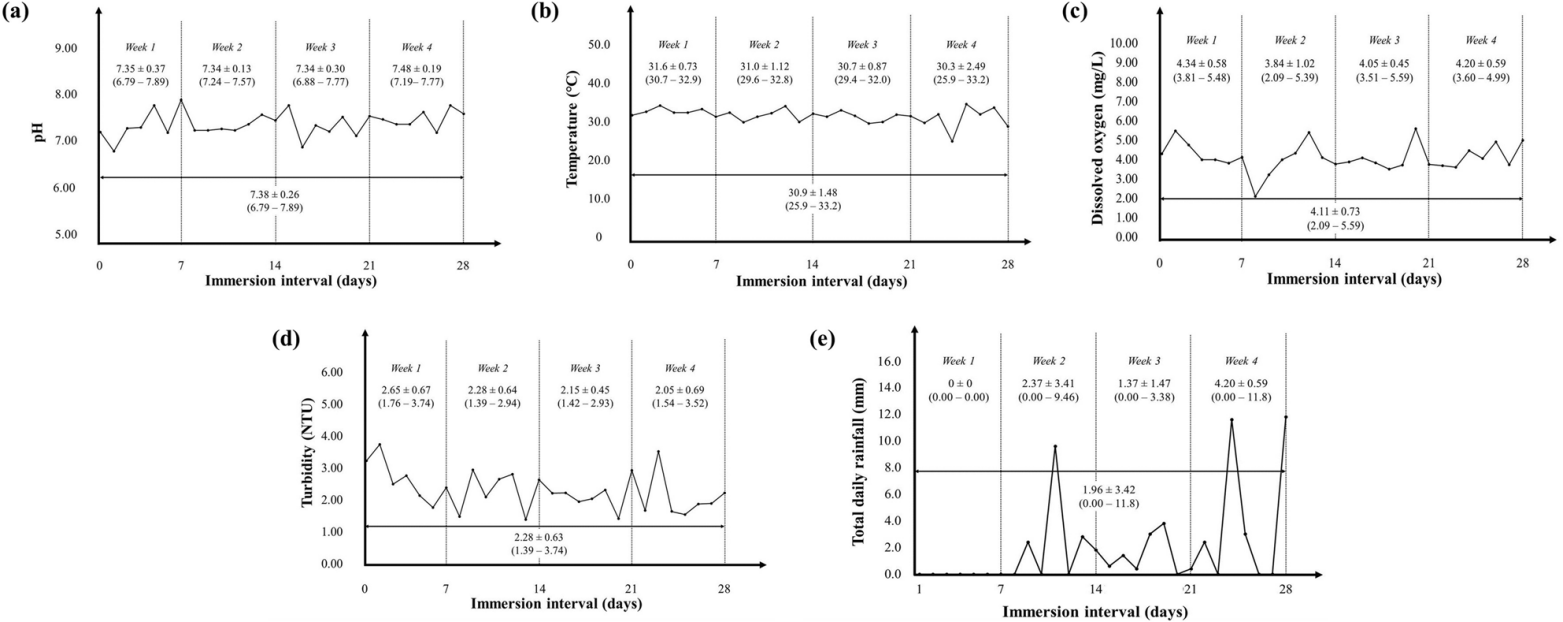
The overall (**a**) pH, (**b**) temperature, (**c**) dissolved oxygen and (**d**) turbidity of the natural outdoor pond water as well as its (**e**) total daily rainfall throughout the four weeks of observation. The data are presented as mean ± standard deviation with values in parentheses () indicate the range.

Since the quality of visualised fingerprints may be affected by physicochemical parameters of water and total daily rainfall, and because the previous research utilising the novel NBR^34,35^ was done under laboratory-controlled settings, fieldwork assessment would prove necessary to elucidate its real practical value. Hence, a natural outdoor pond was chosen for assessing the performance of NBR in visualising latent fingerprints on three different non-porous substrates (i.e. glass slides, laminated plastics and aluminium sheets) immersed for two and four consecutive weeks. The daily measurements of pH, temperature, DO, turbidity as well as total daily rainfall are presented in Fig. 2a–e, respectively.

The overall pH of the natural outdoor pond during the four weeks of observation ranged between 6.79 and 7.89 (mean: 7.38 ± 0.26) (Fig. 2a) with the water being slightly acidic during Weeks 1 and 3. This condition appears consistent with the pH range for a natural pond (5.77–7.74)^39^ and other waterways (5–8)^40–42^ reported by previous researchers. Malaysia being a tropical country with hot and humid weather all year round^43^, the recorded means of daily water temperature of the pond that ranged between 30.3 and 31.6 ℃ were within expectation (Fig. 2b). However, during the observation made on day-24 (Week 4), the water temperature plunged as low as 25.9 ℃ due to the downpour observed (about 11.6 mm). Interestingly, the ranges of pH and temperature of the pond water observed here were invariably within the optimum working pH (about pH 7) and temperature (about 50 ℃) for NBR as reported by previous researchers^44,45^. These conditions were theoretically supporting the use of NBR for fingerprint visualisation on substrates immersed in such a water body for fieldwork assessment.

On the other hand, the range of DO for the pond water was recorded between 2.09 and 5.59 mg/L throughout the observation interval, with the highest mean of DO was recorded during Week 1 (i.e. 4.34 mg/L ± 0.58) (Fig. 2c). In general, the concentration of DO would be inversely proportional to temperature since warmer water can hold less oxygen molecules than cold ones^46^. However, deviation in such aspect was observed at several instances in this present research, probably attributable to increased microbial activities surpassing that of the rate of natural replenishment of oxygen from the air, leading to lower DO values^47^. For instance, the highest recorded DO during the four weeks of observation was on day-20 at 5.59 mg/L, while the recorded temperature of the water was at 31.1 ℃. It has to be acknowledged that there are microorganisms in freshwater that can naturally produce lipases, which in turn may catalyse the lipid constituents of fingerprints on substrates, leading to the difficulty of visualising them. These microorganisms include bacteria (e.g. *Escherichia coli*)^48^ and yeast (e.g. *Candida albicans*)^49^. Considering that the aspect pertaining to the role of lipase-producing microorganisms in the pond was not evaluated here, further research of its influence on the quality of visualised fingerprints merits consideration.

In addition, the turbidity of pond water fluctuated slightly (1.39–3.74 NTU) (Fig. 2d), possibly due to the presence of strong seasonal wind, similar to observation reported by previous researchers^50^. Figure 2e represents the total daily rainfall at the site of the natural outdoor pond throughout the four weeks of observation interval. While the mean amount of total daily rainfall for the whole first week of observation was 0.00 mm, the following weeks saw an appreciable mean amount of total daily rainfall of up to 11.8 mm (heavy rain). Heavy rain might have leeched some of the topsoils along with the edge and plant debris into the pond (surface runoff), resulting in more turbid water. The accumulation of sediments on substrates bearing fingerprints can negatively influence the possibility of visualising latent fingerprints due to reduced accessibility of the NBR and/or SPR towards the lipid and other non-water soluble constituents of the fingerprints respectively. Moreover, the strong mechanical effects of rain and wind can further translate into the more vigorous water current^47^ as a possible physical disruptor of the fingerprints.

#### Quality assessment of the NBR- and SPR-visualised fingerprints using the UC comparative scale

**Figure 3:**
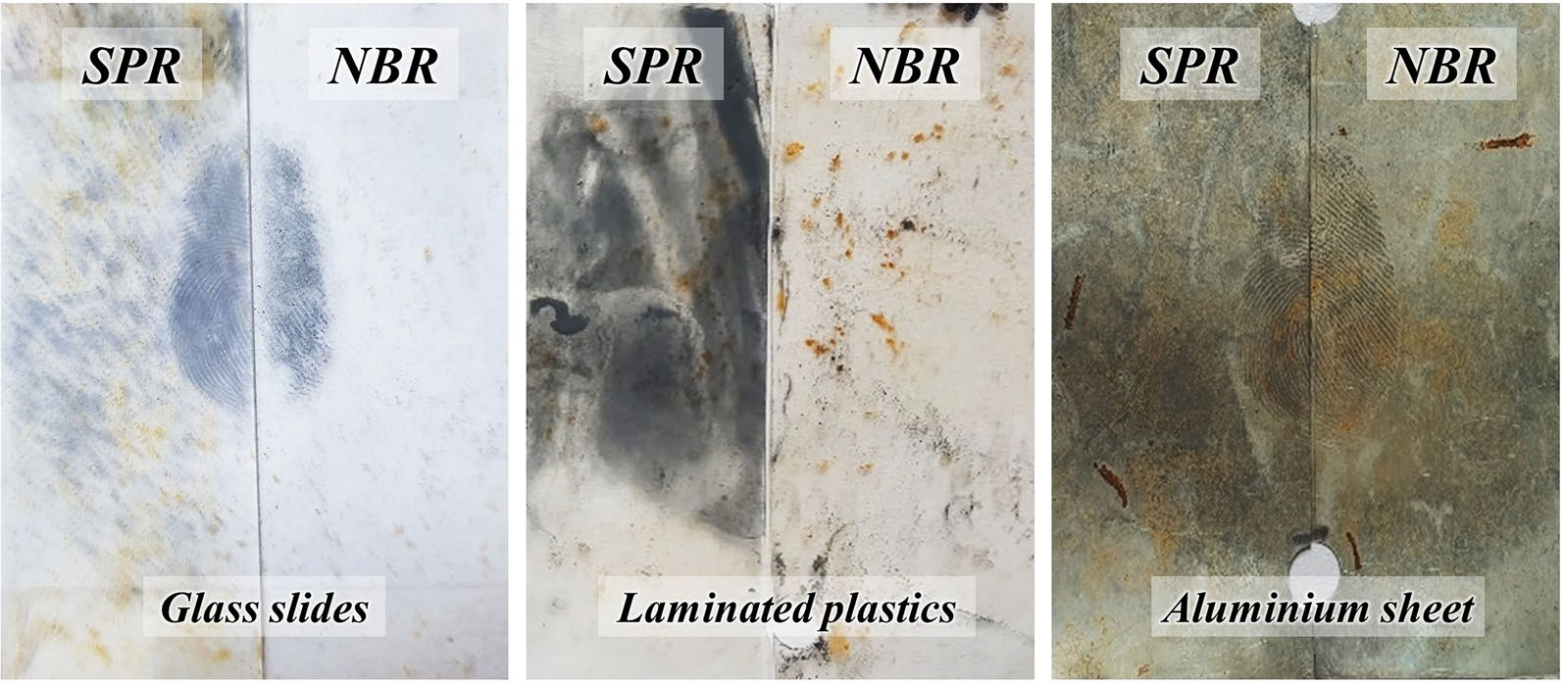
Representative photographs of quality of the visualised fingerprints on glass slides, laminated plastics and aluminium sheets (UC comparative scale: 0) immersed in a natural outdoor pond for four consecutive weeks.

**Figure 4:**
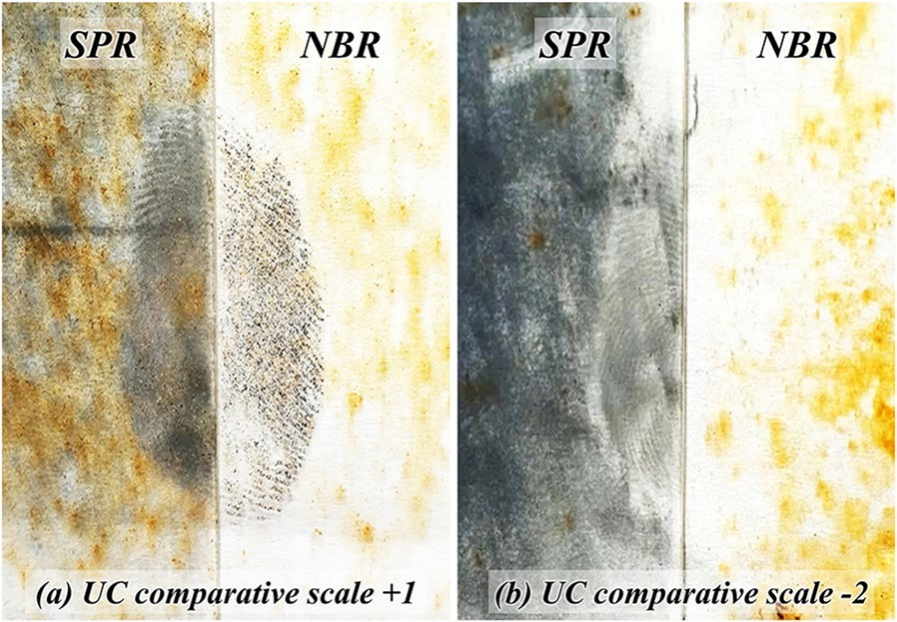
Representative photographs of visualised fingerprints on glass slides immersed for four weeks using both SPR (left) and NBR (right). The sets of split visualised fingerprint depict UC comparative scale of (**a**) + 1 and (**b**) − 2.

The quality of visualised fingerprints (natural and groomed) on non-porous substrates (i.e. glass slides, laminated plastics, and aluminium sheets) immersed for two and four weeks in a natural outdoor pond using the UC comparative scale is presented in Table 2. It is important to note here that for the most part of the two observation intervals, the quality of visualised fingerprints on all three non-porous substrates was at UC comparative scale of 0 (no development), indicating that the performance of this newly-developed optimised NBR was as similar as the benchmark SPR (Fig. 3). It has to be mentioned here that although the UC comparative scale is appropriately useful for assessing the performance of two reagents as suggested by the IFRG^36^, its scale of 0 is not able to indicate if the reagents were able to visualise the fingerprints or vice versa. On that account, development of a more detailed scale for explicitly differentiating such observations may proves necessary. On the other hand, the UC comparative scale of − 1 represented half-impressions developed by SPR exhibited slightly greater ridge detail and/or contrast than the corresponding half impressions developed by NBR. In contrast, UC comparative scale + 1 indicated better performance of NBR in visualising half-impressions than that of SPR (Fig. 4a, b). It is worth mentioning that there were instances whereby the sign of contact was observable although the portion of ridges remained less than 1/3 and discontinuous, with majority of the portion being smudged. Hence, forensic identification of such visualised fingerprints may prove challenging.

**Table 2:** The quality of visualised fingerprints (natural and groomed) on non-porous substrates immersed in a natural outdoor pond for two and four weeks.

Substrate	Donor	Two weeks of immersion						Four weeks of immersion					
		Natural prints			Groomed prints			Natural prints			Groomed prints		
		R1	R2	R3	R1	R2	R3	R1	R2	R3	R1	R2	R3
Glass slides	Female 1	0	0	0	**− 1**	**− 1**	0	0	0	0	** + 1**	0	0
	Female 2	0	0	0	0	0	0	0	0	0	0	0	0
	Female 3	0	0	0	0	0	0	0	0	0	**− 1**	**− 1**	0
	Male 1	0	0	**− 1**	0	**− 1**	0	0	0	**− 1**	** + 1**	** + 1**	**− 1**
	Male 2	0	0	0	0	0	0	0	0	0	0	0	0
Laminated plastics	Female 1	0	0	**− 2**	0	**− 1**	**− 1**	0	0	0	0	0	0
	Female 2	0	0	0	0	0	0	0	0	0	0	0	0
	Female 3	0	0	0	0	0	0	0	0	0	0	0	0
	Male 1	0	0	**− 2**	0	0	0	0	0	0	0	0	0
	Male 2	0	0	0	0	0	0	0	0	0	0	0	0
Aluminium sheets^a^	Female 1	0	0	0	0	0	0	0	0	0	0	0	0
	Female 2	0	0	0	0	0	0	0	0	0	0	0	0
	Female 3	0	0	0	0	0	0	0	0	0	0	0	0
	Male 1	0	0	0	0	0	0	0	0	0	0	0	0
	Male 2	0	0	0	0	0	0	0	0	0	0	0	0

Significant values are in [bold]

^a^Fingerprints were observed on all aluminium sheets even without the use of SPR or NBR. Utilisation of these two reagents neither improved nor reduced the quality of on this kind of substrate. Scale 0: no significant difference between two corresponding half impressions; Scale − 1: SPR performed slightly better than NBR; Scale + 1: NBR performed slightly better than SPR.

In general, both NBR and SPR performed better in visualising the groomed and natural fingerprints on glass slides than on laminated plastics. The observations were in concurrence with that observed during the stability and sensitivity assays reported in our previous publication^34^, perhaps because glass slides have a less-slick and shiny appearance that had permitted the available chemical constituents of fingerprints to remain on the surface longer than the ones on the slicker laminated plastics. It was observed that the NBR performed slightly better than that of SPR in three occasions involving aged, groomed fingerprints (Week 4) on glass slides, revealing the values of + 1 (Fig. 4a). These NBR-visualised fingerprints had 1/3–2/3 continuous ridges; therefore, forensic identification was possible. On the other hand, the SPR had performed slightly better than that of NBR in eight occasions; One and two instances of − 1 and − 2 for natural fingerprints as well as five instances of − 1 for groomed fingerprints immersed for two weeks in the pond, respectively. Assessment of the quality of these SPR-visualised fingerprints revealed that they were also of 1/3–2/3 continuous ridges (Fig. 4b), similar to the modified Centre for Applied Science and Technology (m-CAST) absolute scale of 2, whereby forensic identification was indeed possible.

The results also demonstrated variations in the quality of fingerprints visualised using both NBR and SPR (Table 2) despite the fact that the fingerprints were deposited on similar substrates and immersed in the same pond for the same intervals (two and four weeks). Such variations were generally expected since studies have shown inter-variability in the constituents of fingerprints among the different donors. Factors influencing such variations can be categorically divided into characteristics of the donors (e.g. diet, age and gender) as well as conditions when depositing the fingerprints (e.g. pressure and duration of contact)^7^. Considering that differences in diet among donors, as well as pressure and the maximum contact time for depositing the fingerprints were not standardised here, further research factoring such aspects deserves considerations.

While both NBR and SPR failed to visualise most of the fingerprints on the glass slides and laminated plastics satisfactorily, it can be observed that the NBR had higher preference towards aged, groomed fingerprints (Week 4) than that of the fresher as well as natural ones. In contrast, SPR favoured the natural fingerprints over groomed and aged ones. The observed preference of NBR towards the aged and groomed fingerprints can be explained by the fact that lipases are specifically detecting lipids^51^. On fresher/dry fingerprints, the lipids may be hindered by other constituents (e.g., proteins, wax esters, squalene, etc.), restricting the accessibility of the lipases. This is further supported by the inability of NBR to visualise latent fresh and dry fingerprints, as observed during the preliminary investigations. Over time, such constituents may have been cleared up by water, thereby allowing access to the NBR towards the lipid constituents of fingerprints and enabling visualisation. The less preference of NBR towards the natural fingerprints may be attributable to the limited amount of lipids available on fingerprints^7^ and hence, does not support its visualisation. Remarkably, less background interferences were observed on all NBR-visualised fingerprints (Fig. 3) which can be attributable to the high specificity and selectivity of CRL towards the lipids^52^. This observation was also consistently seen in our previously reported studies^34,35^. As for the SPR, the mode of interaction for the visualisation of fingerprints on wet, non-porous substrates remains unknown^23^; thus, suitable discussion on its preference on the non-water soluble constituents of fingerprints proves limited.

It is also worth noting here that significant degradation in the quality of fingerprints was observed on all samples, consistent with its increased prolonged immersion in the natural outdoor pond. Pertinently, most of the samples suffered marked background interference (covered in thick mud) and thereby, further complicating the visualisation process. Since immersing them in the pond can be quite destructive (possibly due to the underwater microbial activity that may hydrolyse the lipid constituents of fingerprints as well as the mechanical effect of the water itself), such an observation was within expectation. This finding too was in concurrence with other previously reported studies^53,54^.

#### Analytical Eco-Scale for assessing the greenness of the novel NBR

The concept of analytical Eco-Scale to semi-quantitatively determine the greenness level of certain processes has been widely advocated in green analytical chemistry^38^. Although the concept originated from organic synthesis processes^55^, its utilisation for the preparation of fingerprint visualisation reagents appears nascent. The Eco-Scale metrics would prove useful for identifying the weakest points in the methodology by comparing the different parameters and steps to meet the requirement for green chemistry^38^. In this context, the adaptation of such a metric for categorically assessing the greenness scale of the newly-developed NBR would pave the way for facilitating suitable future discussions in green fingerprint visualisation technology development. Table 3 represents the calculated analytical Eco-Scale for the NBR.

**Table 3:** The penalty points for preparing the NBR for fingerprint visualisation.

Currently-developed NBR	PP	Previously-developed reagent (Azman et al*.*, 2018)	PP
**Chemicals**		**Chemicals**	
Hydrochloric acid^a^	2	Hydrochloric acid^a^	2
Nitric acid^b^	4	Nitric acid^b^	4
Sulfuric acid^c^	2	Sulfuric acid^c^	2
Multi-walled carbon nanotubes^d^	2	Multi-walled carbon nanotubes^d^	2
*Candida rugosa* lipase^e^	0	*Candida rugosa* lipase^e^	0
Phosphate buffer (pH 7)^f^	0	Phosphate buffer (pH 7)^f^	0
		Glutaraldehyde^g^	8
		Safranin T dye^h^	2
	**Σ** **10**		**Σ** **20**
**Instrument**		**Instrument**	
Hot plate	2	Hot plate	2
Oven	2	Oven	2
Centrifuge	0	Centrifuge	0
Fridge	2	Fridge	2
	**Σ** **6**		**Σ** **6**
Occupational hazard	3	Occupational hazard	3
Waste	5	Waste	5
	**Σ** **8**		**Σ** **8**
**Total penalty points**	**24**	**Total penalty points**	**34**
**Analytical eco-scale total score (100–24)**	**76**	**Analytical eco-scale total score (100–34)**	**66**

Preparation of F-MWCNTs as described by the USains Holdings Sdn Bhd (7 M HNO_3_:H_2_SO_4_ (1:1) refluxed for 3 h at 90℃).

^a^(Merck, 2018a),^b^(Merck, 2020),^c^(Merck, 2018b),^d^(Sigma-Aldrich, 2020),^e^(Sigma-Aldrich, 2012),^f^(QRec, 2011),^g^(Sigma-Aldrich, 2019) and ^h^(Sigma-Aldrich, 2016).

The calculated analytical Eco-Scale for NBR was 76, fell well within the prescribed value for indicating ‘excellent green analysis’ (≥ 75)^38^. In contrast, greenness Eco-Scale calculation for the previously developed method utilising immobilised lipases as fingerprint reagent^54^ scored 66, which can be categorised as the ‘acceptable green analysis’ (˃ 50). Therefore, this newly-developed NBR was relatively greener than the previous visualisation method that we developed^54^. This reagent did not require the use of hazardous chemicals like glutaraldehyde and safranin T dye. The use of both glutaraldehyde and safranin T dye solutions described by Azman et al*.*^54^ added an additional ten penalty points (PP) to the Eco-Scale calculation. While it can be construed that the NBR is an effective and green reagent for forensic fingerprint visualisation on wet substrates, further improvements can be made by reducing the amount of waste since the immobilisation of CRL onto F-MWCNTs may result in its enhanced reusability as well as the stability of the enzyme^56,57^. Meanwhile, suitable analytical Eco-Scale comparison with SPR was not done here considering that would require restricted information from manufacturer (e.g. the required energy used and generation of waste during the SPR preparation process).

#### Possible forensic applications of the novel NBR

Having considered the overall findings, recommendations can be made to the forensic investigation team on the sort of potential exhibits (i.e. metal sheets and glass) to be collected in aquatic investigations since the visualisation of fingerprints was completely unsuccessful for laminated plastics. This is to maximise the possibility of securing suitable fingerprints for identification purposes and optimise laboratory resources for forensic practical caseworks. However, further investigations utilising larger sample sizes on various substrates as well as immersing them in different types of water exposures with diverse physicochemical parameters may be required to clearly elucidate their real forensic values. Secondly, the fact that the NBR preferred fingerprints at a longer immersion period, its usefulness for visualising fingerprints on underwater evidence recovered up to 4 weeks of immersion appears empirically supported. This is particularly useful in crime situations where evidence like murder weapons and glass fragments are discarded in similar aquatic environments. The fact that the reagent remained stable even at 45 ℃^34^, its practicality for field work applications in hot and humid countries like Malaysia is justifiable and advantageous. In this context, it is pertinent to cite a statement made by Kent^58^ that “We would all like to see more effective fingerprint development techniques, particularly for some of the more difficult surfaces, but it is essential that we are not seduced into giving up well-tried and documented methods by the superficial attraction of a “new” technique until we have reliable data.”. Therefore, researchers should not be seduced by the new technology reported at the expense of the currently well-established techniques, but rather we should explore the possibility of improving forensic technology by complementing the existing ones. Should the NBR developed here be incorporated into the practice, it can be considered as one of the alternatives rather than explicitly removing SPR from practice. However, we also highlight several aspects such as the incomplete understanding of the mode of action for SPR that rendered lesser development and improvement to the technology as reported in the literature. The fact that we explored the suitability of enzymology for use as well as the suitable explanations on the mode of interactions^35^, this piece of information would serve as stepping stones to further explore enhancement processes that can be imposed so that improvements to the overall fingerprint visualisation technology can be made.

## Conclusion

In this present research, a novel optimised green nanobio-based method was successfully evaluated against the routinely-used SPR for the rapid visualisation of latent fingerprints on wet, non-porous substrates immersed in a natural outdoor pond. Taking into account the exigent need to visualise wet latent fingerprints for forensic identification purposes as well as because the commercially-available SPR is associated with numerous toxicities towards both humans and the environment, the evaluation of green optimised NBR against the SPR was therefore warranted. In general, the forensic assessment of the NBR revealed its comparability against the benchmark SPR (UC comparative scale: 0) in visualising fingerprints on all three substrates (i.e. glass, laminated plastics, and aluminium sheets) immersed in a natural outdoor pond for up to four consecutive weeks, with glass being the preferred substrates for both natural and groomed fingerprints. Noticeably, the NBR had a higher preference in visualising fingerprints immersed at longer period, and therefore, demonstrating its practicability for forensic investigations considering that underwater evidence is normally recovered long after the crime was committed. On the other hand, the greenness assessment of the NBR resulted in a total score of 76, signifying ‘excellent green analysis’ and thereby further supporting its practical use for on-site crime investigations. Given the overall findings of this present research, the NBR evaluated here could potentially be the future state-of-the-art green fingerprint technology in forensic investigations. Notwithstanding, it is important to indicate here that this present research is considered as partly at Phase 2, referring to the guidelines published by the IFRG^36^. In order for the reagent to be acceptable for use, it needs to undergo two more phases of assessments (Phases 3 & 4) as stipulated in the said guidelines.
